# Developing a Mass Spectrometry-Based Research and Teaching Program at a Primarily Undergraduate Institution

**DOI:** 10.1021/jasms.5c00180

**Published:** 2025-08-05

**Authors:** Jay G. Forsythe

**Affiliations:** Department of Chemistry and Biochemistry 2343 College of Charleston 66 George Street, Charleston, South Carolina 29424, United States

**Keywords:** primarily undergraduate institution, undergraduate, mass spectrometry, analytical chemistry

## Abstract

Primarily undergraduate institutions (PUIs) play critical roles in the higher education framework of the United States. In this Perspective, considerations for establishing a mass spectrometry (MS)-based research and teaching program at a PUI are discussed. Topics covered include balancing teaching, research, and service responsibilities, acquiring MS instrumentation, and establishing a sustainable research program for undergraduate students.

## Graphical abstract

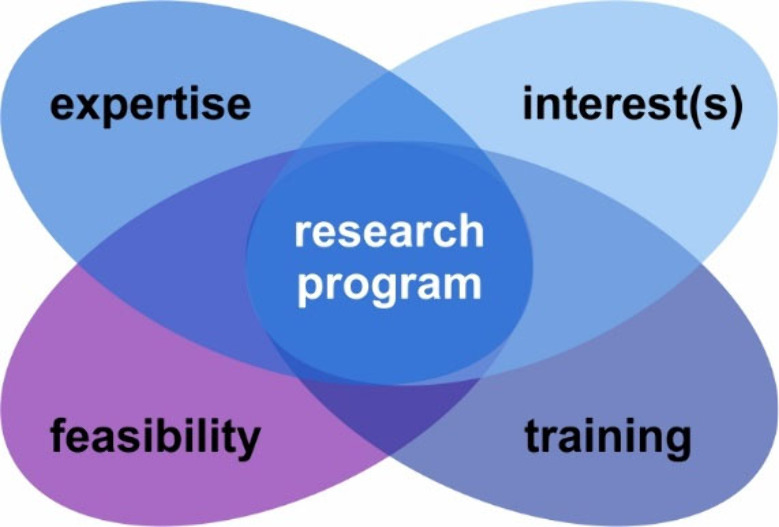

## Introduction

Primarily undergraduate institutions (PUIs) contribute to STEM education, research, and workforce development in the United States. PUIs vary in type and size, and include but are not limited to liberal arts colleges, some historically black colleges and universities (HBCUs) and minority serving institutions (MSIs), and numerous public colleges and universities.^1–5^ By definition, PUIs either do not have doctoral programs or their doctoral programs are very limited. At the time of writing, the National Science Foundation (NSF) defines “non-Ph.D.-granting institutions” as those which award less than 20 doctorates in supported fields in a two-year period.^6^

PUI research infrastructure is less than at Ph.D.-granting institutions. Nevertheless, PUI faculty can do impactful work. Mass spectrometrists such as Elaine Marzluff at Grinnell College,^7–9^ J.C. Poutsma at the College of William and Mary,^10–13^ Christine Hughey at James Madison University,^14–16^ and Jim Pesavento at St. Mary’s College (CA)^17,18^ —just to name a few—have contributed notable findings to our field while working with undergraduates.

In this Perspective, I describe my experiences at a PUI over the past nine years and provide advice for those interested in developing a mass spectrometry (MS)-based research and teaching program for undergraduates. As with any job, working at a PUI has its challenges. The main challenges I have faced to date are (1) balancing teaching, research, and service, (2) acquiring MS instrumentation, and (3) establishing a sustainable research program. These are discussed below.

## Balancing Teaching, Research, and Service

### Teaching

I work at a public liberal arts institution of ∼12,000 students (>90% undergraduate, ∼20% underrepresented minorities). My department does not have any graduate students. I mainly teach general and analytical chemistry courses and occasionally teach specialty courses such as first-year seminars or upper-level research classes. I have three courses each semester (a.k.a. a 3-3 “teaching load”). Lecture and lab courses are listed separately at my institution but count equally in terms of faculty teaching load; this is not true for all PUIs. Based on conversations with faculty at similar research-active PUIs, a 3-3 teaching load seems about average.

Teaching is an essential part of life at a PUI. I spend much of my time during the academic year teaching lecture and lab courses, preparing course materials, meeting with students, and grading assignments. It is challenging to build materials for a new course—early on, I leaned on more established faculty for advice and help. After a few times through a class, it gets easier. It is important to update course materials regularly in analytical chemistry, but this is less demanding than starting from scratch. A few books on college teaching that were helpful to me are *Teach Students How to Learn* by retired chemist Saundra Yancy McGuire,^19^ *What the Best College Teachers Do* by Ken Bain,^20^ and *Small Teaching* by James Lang.^21^ More specifically, when it comes to teaching MS, I have found recent articles from Amanda Patrick (Mississippi State) and Matthew Joyner (Pepperdine) useful.^22,23^

We do not have teaching assistants (TAs) in my department. Grading takes up a lot of time during the academic year, including some nights and weekends. In introductory courses, it is necessary to uphold departmental norms and expectations regarding content and assessment. Upper-level and specialty courses are more flexible; I often use specifications-based grading for these.^24,25^ Briefly, specifications grading involves clear, tangible learning outcomes evaluated on a pass/fail basis with opportunities to resubmit or reattempt. I use this approach to evaluate both writing assignments and hands-on laboratory skills (e.g., tryptic digestion of a protein into peptides).

Perhaps a reader is thinking: “Would I be a good teacher?” or “Being in front of an entire class must be scary.” It is normal to be nervous about teaching—these fears tend to subside with time and experience. I will never forget starting out as a TA in graduate school. I was terrified. By the end of my first year, I loved teaching and wanted to work at a PUI someday. Both my Ph.D. and postdoctoral advisors were supportive of this career goal and provided opportunities for me to teach as a trainee. I was nervous again when teaching my first few courses at College of Charleston but, once more, the nerves subsided with time.

I think the best way to learn how to teach is, simply, to teach. Much of what I have learned about teaching has come by trial and error. Departmental colleagues have been extremely helpful as well. Several have observed my lectures to provide feedback and write letters of evaluation used for tenure and promotion. I keep an eye on chemical education literature (e.g., *J. Chem. Ed*.) and value student input also. I have received some wonderful insights from students over the years, most of it via direct conversation. I take course evaluations with a grain of salt—it is well-known they are flawed evaluation tools.^26,27^ My teaching priorities are to encourage students to work hard, treat them with fairness and empathy, maintain healthy boundaries (acknowledging power dynamics between faculty and students), and show enthusiasm. No teacher is perfect. *Wanting* to improve is what matters.

### Research

Teaching and research are deeply intertwined at a PUI. When I am in the research lab with students, I am teaching. Likewise, research keeps me up-to-date with the field and improves my instruction. I have spun out new courses based on my research; this is quite common at PUIs. When opportunities arise to further blend teaching and research, I typically say “yes.” For example, in 2020, I developed a first-year writing seminar focused on the origins of life and search for life elsewhere in the solar system, which is related to my research program.

Mentoring undergraduates in research is my favorite thing about the job. I am continually inspired by the eager, bright young scientists with whom I work. I have mentored or comentored 44 students to date: 19 undergraduates in my group (∼60% women, ∼30% from underrepresented minorities), 22 undergraduates in upper-level research courses (discussed below), and 3 visiting students through a German exchange program. Our group typically has 3–5 students at any time. Students work in the lab most of the academic year and for 10 weeks in the summer. Breaks are good times for instrument maintenance, catching up on writing, or perhaps a much-needed vacation!

During the academic year, research progress can be slow. Everyone is busy with classes. Many of our students work part-time as well. Students receive credit for research during the semester and work in the lab anywhere from 3 to 10 h per week. I try to recruit new lab students early in their college experience, ideally in their first year or by the fall of their sophomore year.

One way to make gains during the school year is to teach a course undergraduate research experience, or CURE.^28–30^ These upper-level courses are centered on active research projects; student assessment may involve research skills gained, making/presenting a poster, giving an oral presentation, or writing a paper. In the spring of 2025, a colleague and I cotaught a CURE which focused on peptide synthesis, characterization, and sequencing by tandem MS (MS/MS). The students solved several problems we had been facing in our collaborative project and gave a poster at a school-wide symposium.

Summer is the most focused time for research. Our undergraduate researchers work ∼40h per week in the summer. I am in the lab with them for the bulk of this period, teaching how (and why) to do experiments, troubleshooting instruments, interpreting data, and so on. Summer research goes by fast, especially when the group attends a conference such as ASMS. I encourage all of my research students to present work at a conference; these are highly valuable experiences and strengthen their CV for graduate or job applications.

After a few semesters and/or a summer in the lab, students grow in their independence. They can be quite productive in subsequent semesters, and very productive if they stay in the lab for a second summer. Seniors from our Honors College are required to write research theses before graduation; these are excellent starting points for publications. There have been times when I had to reevaluate student data before submitting a paper or go into lab to finish up a project myself. A colleague called this role “super-postdoc,” which I think is apt.

### Service

Service is a catch-all term for the various professional activities of a faculty member not considered teaching or research. I view service at a PUI to be in three categories: service to students, service to the department/institution, and service to the field/profession.

PUI faculty serve students through academic advising, connecting them with various research opportunities or internships, writing letters of recommendation, providing direction to helpful resources on campus, etc. I spend a lot of time advising students, getting them in touch with campus resources, and writing recommendation letters.

Service to one’s institution can include things like faculty-wide committees, reviewing job applications, departmental curriculum reviews, or participation in faculty senate. Some of these are not optional (e.g., reviewing job applications), and some are. I currently serve on our Faculty Research and Development committee and as Campus Director for the South Carolina NASA EPSCoR/Space Grant program. Additionally, PUI faculty may have responsibilities in maintaining shared-use analytical instrumentation. I am fortunate in this regard—we have an excellent Ph.D.-level instrument manager. I help out with the mass spectrometers but do not perform other routine maintenance.

External service and outreach opportunities—reviewing papers and grants, involvement in professional societies, organizing a session at a meeting, etc.—remind me that I am not only a teacher but also a scientist. In the past, I participated in ASMS activities such as the Undergraduate Research in MS interest group (2018–2020) and hosting the Historic Replica (2020–2021). Regarding reviews, I accept about 2–3x more manuscripts/grants than I submit myself, prioritizing journals and topics related to my research. I encourage early career faculty to participate in grant review panels—these positively impact the scientific community and provide insights into how to write better proposals. Some NSF programs to consider are Major Research Instrumentation (MRI; discussed later) and Graduate Research Fellowship Program (GRFP). It is not presumptuous to contact a Program Officer and express willingness to participate.

Service is expected for tenure and promotion but is less significant than teaching and research. I prioritize student service and then find a healthy balance between institutional and external service. A colleague noted that analytical chemists tend to be recruited for service tasks (and, later, administrative roles). Early career faculty should be cautious about doing too much service. I have said “no” to a number of service opportunities in order to say “yes” to those that best aligned with my interests, strengths, and time.

### The PUI Balancing Act

At least for me, the greatest challenge about working at a PUI has been juggling various responsibilities and making progress on multiple fronts. Over a year, I spend about 45% of my time on teaching, 45% of my time on research, and 10% of my time on service. However, my day-to-day experience fluctuates greatly. In November, I spend 75–80% of my time on teaching activities. In June, I spend 95–100% of my time on research. I have improved much in my time management skills but still have plenty of room to grow.

Mentorship regarding time management is important. Some departments/institutions have a formalized mentorship program and others do not. Regardless, one can always seek out mentors. Scientists are good at observation; I would try to find a mentor or two and watch how they go about their day.

During the school year, it is easy to fill a day with short-term tasks (grading papers, answering emails, serving on committees, etc.) that do not impact one’s long-term professional development or research program. I have started to block off time for tasks that cannot be done quickly but are important, such as outlining a grant or designing a new lab course.

## Acquiring MS Instrumentation

Mass spectrometers are expensive, and PUI start-up funds are significantly less than at large universities. It is possible to collaborate with researchers at larger universities to run samples and acquire data for publications or grants, which is addressed briefly in the following section. Additionally, some federal laboratories have competitive application processes for external users to request free time on their instruments.^31^

Nevertheless, I think it is a worthwhile pursuit to acquire instrumentation and build MS capacity at one’s home institution. I want our undergraduates to get hands-on training with mass spectrometers and build interest in analytical science. Mass spectrometers are, more or less, shared at a PUI; new acquisitions can boost research and teaching activities for an entire department or school. Also, on-campus instrumentation can help to recruit new faculty. When I was on the job market in 2015, I was acutely aware that College of Charleston had an LTQ Velos Pro linear quadrupole ion trap (LQIT) MS, obtained via NSF MRI funding (Table 1).

**Table 1:** Current and Recently Retired MS Instrumentation in the Department of Chemistry and Biochemistry at College of Charleston^a^

Type	Model	New?	Installed	Retired?	Obtained via
LC-MS (QIT)	LCQ Advantage Max	Y	2007	2018	NIH-INBRE (S. Carolina)
GC-MS (Q)	7890*A*/5975C	Y	2010		Dept. funds
LC-MS (LQIT)	LTQ Velos Pro	Y	2013		NSF MRI (2012)
MALDI-TOF	Voyager DE-STR	N	2017	2023	Start-up funds
GC-MS (Q)	Trace 1300/ISQ 7000	Y	2021		Dept. funds
nanoLC-LQIT/Orbitrap	Orbitrap Elite ETD	N	2023		Private donation
MALDI-TOF/TOF	autoflex maX	Y	2024		NSF MRI (2023)
LC-MS (QQQ)	TSQ Quantum Max	N	2025		In-state donation

a Mass spectrometers are listed in chronological order of acquisition.

As a postdoctoral fellow, I mainly used ion mobility (IM)-MS and LC-IM-MS/MS instrumentation.^32,33^ However, when I started out at College of Charleston in 2016, I thought matrix-assisted laser desorption/ionization–time-of-flight (MALDI-TOF) would be more feasible for my group, and a complementary teaching tool to the LTQ Velos Pro with electrospray ionization (ESI). My start-up package was reasonable for a PUI at the time, but buying a new instrument was out of the question. I needed to allocate most of the funds for basic equipment and supplies such as an analytical balance, deionized water system, pipettes, solvents, and so on. I stocked the lab and looked into cheap, refurbished MALDI-TOFs.

I settled on a Voyager DE-STR that was originally built in 2000 but had refurbished turbomolecular pumps, detectors, and a nitrogen-gas 337 nm laser (Figure 1a; Table 1). I chose it for the following reasons: (1) I had used a Voyager in graduate school and was quite familiar with its operation and maintenance; (2) used parts were available; (3) it was robust and suitable for undergraduates; and (4) it was *cheap*—the price of a car, not of a house. I had to fill out “sole-source” paperwork to bypass a bidding process and get the Voyager from a specific vendor I trusted. The paperwork was, frankly, a nightmare. It took months to purchase the Voyager. It was installed just in time, one week before summer research.

**Figure 1:**
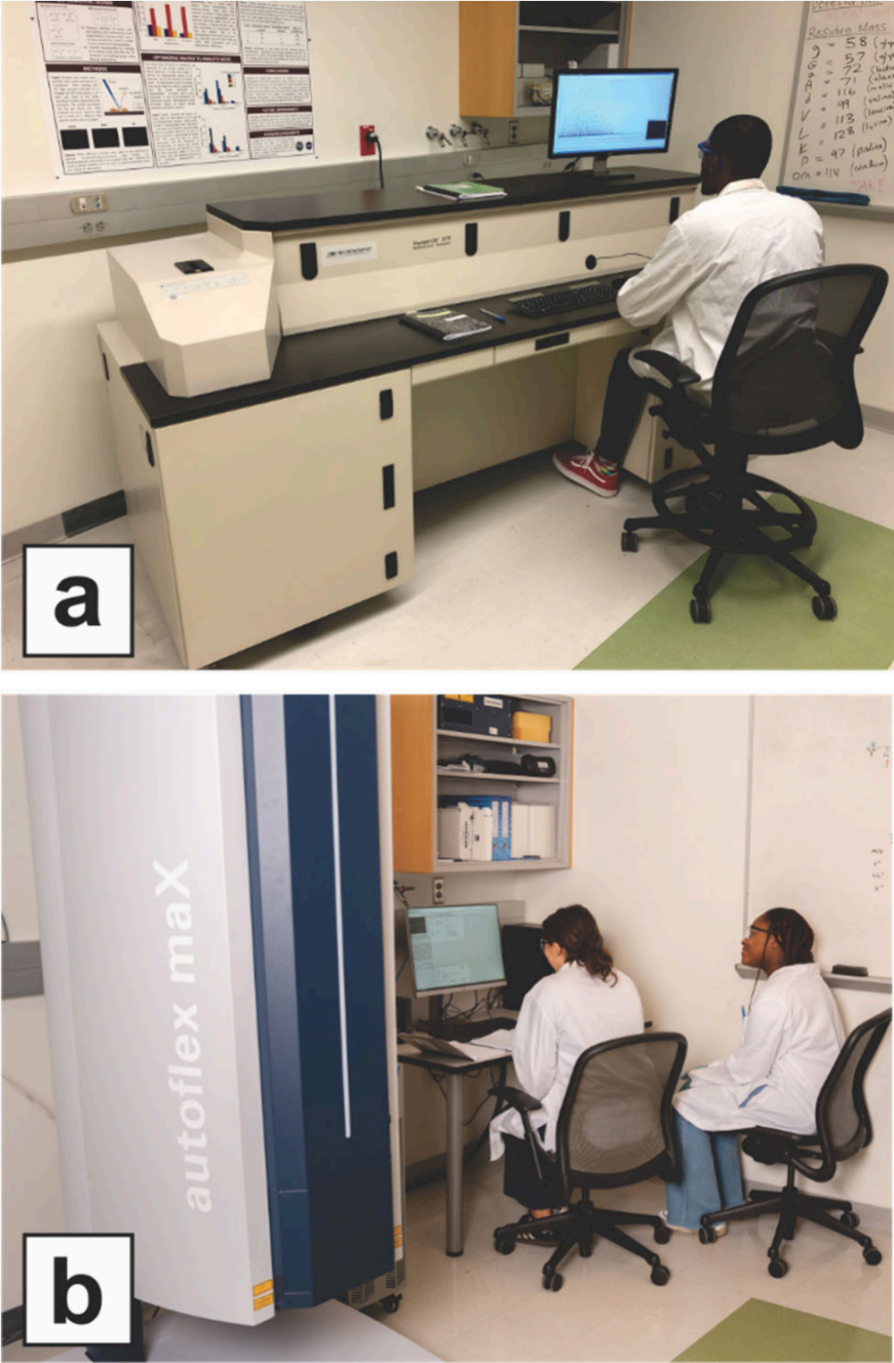
Old and new MALDI-TOF instruments in our lab. (a) Voyager DE-STR mass spectrometer with an undergraduate student operator. Instrument was refurbished in 2016, installed in 2017, and retired in 2023. (b) New autoflex maX MALDI-TOF/TOF mass spectrometer with two undergraduate student operators. Instrument was installed in early 2024.

Despite the stress of getting the Voyager, it was just what I needed. Data from that first summer of research resulted in two publications with undergraduate coauthors^34,35^ and multiple student presentations. Its usage in our department grew rapidly, including in Instrumental Analysis and Bioanalytical Chemistry lab courses. The Voyager was used by ∼150 persons in six years, including seven Goldwater Scholarship recipients. Voyager data was featured in nine peer-reviewed manuscripts with 18 undergraduate coauthors. Despite its age, it was quite robust. I only had to replace a few parts (turbo pump, ion gauges, vacuum control box) over six years. However, by 2023, multiple components were failing at the same time, so we submitted an NSF MRI grant to replace it with an autoflex maX MALDI-TOF/TOF (Table 1; Figure 1b). The grant was funded, and we installed the new instrument in early 2024. According to the reviews, the old age of the Voyager, its heavy usage, and preliminary data directly comparing the two models strengthened the case for a new MALDI.

I have plenty of experience with unsuccessful NSF MRI proposals also. In fact, the 2023 grant was my fifth attempt at MRI funding, but my first for a MALDI. The previous four attempts were for a high-resolution LC-MS. In those proposals, we tried to argue that high-resolution, accurate mass analysis would advance research and teaching at our institution. Some proposals were well-reviewed but not funded, and others were not discussed in panel. In hindsight, the underlying thread of panel and reviewer comments—that we did not justify all projects and teaching examples actually needed high-resolution, accurate mass measurement—were correct. We had the LTQ Velos Pro LC-MS already, and it was sufficient for a number of projects. I (eventually) learned my lesson and shifted focus to a new MALDI.

I encourage early career faculty to write NSF MRI proposals when able. Despite tough competition (even among other PUIs), it is the best route to acquire a new mass spectrometer. Upgrading from the Voyager to the autoflex maX has enabled new research and teaching directions that were not possible before, even when the Voyager was in good condition.

Another way to acquire an instrument is through donation. Instrument donations are typically secured through professional networks—former mentors, industry connections, nearby institutions, etc. In 2023, a colleague (and co-PI on our unsuccessful LC-MS proposals) secured a 10-year-old Orbitrap Elite (Table 1) from a private research university that was upgrading to a new model. They had kept the Elite under service contract for its entire lifetime. (It is no longer under contract with us.) We now have high-resolution, accurate mass capabilities on campus and are using this instrument primarily for research. In 2025, a triple quadrupole (QQQ) was donated from a nearby public institution; at the time of writing, we are getting it up and running (Table 1).

I would be shrewd when it comes to used instruments, however. Mass spectrometers do not last forever, and models vary in robustness and cost to repair. I am particularly wary of instruments that have been inactive for long periods. Salvaging old instruments can be fun and educational but, in most cases, should wait until after going up for tenure and promotion. I recommend starting out with something that works.

## Establishing a Sustainable Research Program

My research group works at an intersection of MS, analytical chemistry, and astrobiology. We use MS, and develop MS-based methods, to study peptides and depsipeptides—copolymers of amino acids and hydroxy acids—in the contexts of chemical evolution and possible biosignature detection in the solar system.^36,37^ (We use infrared spectroscopy to a lesser degree also.^38^) I started in this field as a postdoctoral fellow in the Center for Chemical Evolution and continued to collaborate with the group after I moved to College of Charleston. Currently, my lab collaborates with several colleagues on campus and two NASA centers. I attend analytical conferences such as ASMS and Pittcon and also astrobiology meetings such as AbSciCon. I enjoy engaging with both professional communities. There are others that work in this area too, but it is a relatively small group.

I have tried to carve out a research niche that can draw interest from people (students, colleagues, administrators, reviewers, etc.) but is not overly competitive. Projects in my group take 3–5 years and span multiple undergraduates, who are often a year or two out of college by the time their work is published. I cannot compete against laboratories full of graduate students, postdoctoral fellows, and million-dollar instruments. I prefer to collaborate than to compete, working with people who value PUI training and understand that things can move slowly. With everyone on the same page, such partnerships can be mutually beneficial (Table 2).

**Table 2:** Collaboration between PUIs and Ph.D.-Level Institutions/Federal Labs Can Be a Win-Win for Both

Benefits to PUIs	Benefits to Ph.D.-level institutions/federal laboratories
Expertise of Ph.D.-level lab investigator/their group	Expertise of PUI investigator/their group
Increased speed/scale/impact of a project	Recruitment of students for grad programs/internships
Access to advanced instrumentation	Mentorship opportunities for grad students/postdocs
Student placement in grad programs/internships	Supplemental mentorship of PUI investigator to group
Student exposure to postgraduate/professional laboratories	UG student labor (e.g., summer pay) is inexpensive
Build external reputation of PUI institution	Increased competitiveness of grants (e.g., NSF)
Potential site for PUI investigator to do sabbatical	Collaboration is a priority to PUI (due to limited time)

Several factors come into play when thinking about what to research at a PUI. In my opinion, the main ones to consider are expertise, interests, desired student training, and—not least of which—feasibility. A Venn diagram is shown in Figure 2, along with key questions to ask oneself when considering new ideas. A research program is never etched in stone; one can always change directions. I shifted the goals of my research program during a sabbatical in 2023–2024; new ideas came about through working on grants with collaborators.

**Figure 2:**
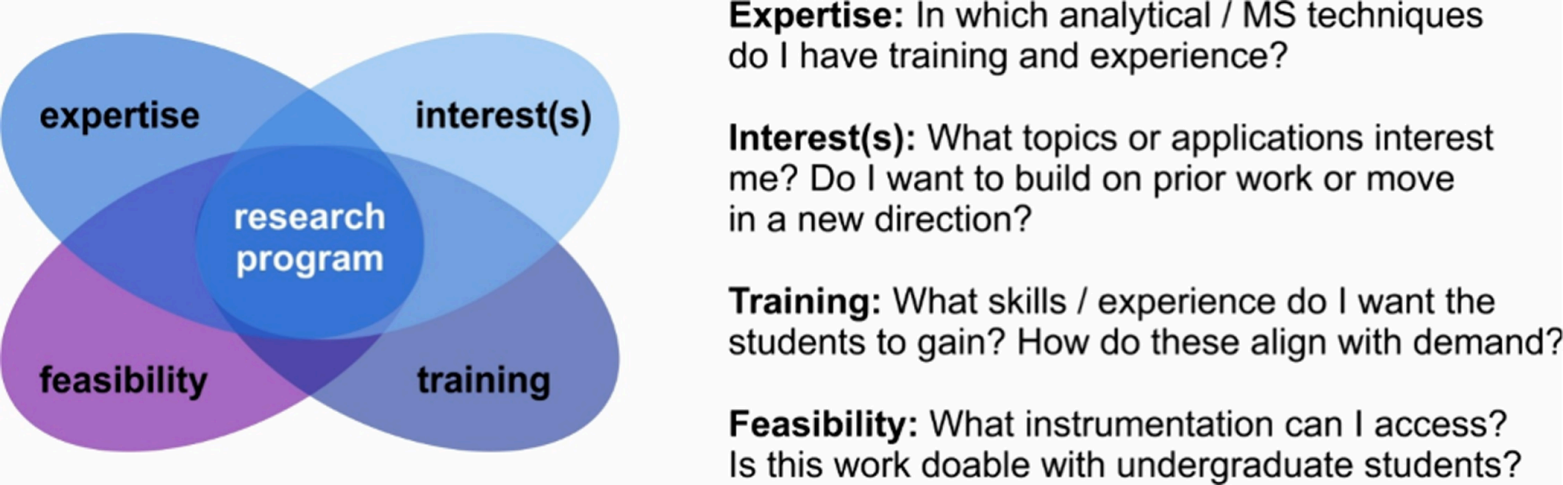
Venn diagram of key factors in developing a PUI research program: expertise, interest(s), training, and feasibility.

It is also important to find sustainable sources of funding for personnel, supplies, and travel. Undergraduate researchers do not cost nearly as much as graduate students or postdoctoral fellows; usually, they are paid in the summer only. Still, it is necessary to plan in advance how, and how much, students will be paid during the summer. Some may be willing to do research for free, but this is not equitable. Most have to choose between research or a job.

My institution has several internal funding sources for summer research, and I have used them between external grants or if I accepted more students in my group than had spots on a grant. Some PUIs have endowments specifically for research and related costs. A good question to ask on a PUI job interview is “How are undergraduates typically paid at this institution for summer research?” Schools invest in what they value.

Many PUI faculty are on 9- or 10-month contracts. Additional summer pay for new faculty can be negotiated as part of start-up, but sustained summer pay for faculty typically requires external support.

At the time of writing, the funding climate is quite uncertain. I think it is wise to diversify proposal submissions both in terms of agency and grant size. In addition to institutional funds and federal grants, there are grants at local or state levels, grants and awards from professional societies such as ASMS and the American Chemical Society (ACS), and private foundations which fund research.

Small grants can go a long way at a PUI. It is entirely possible to sustain a PUI research program without consistently getting large grants. In recent years, I have funded my group through a combination of Established Program to Stimulate Competitive Research (EPSCoR)^39^ and NASA grants. If one’s institution is in an EPSCoR state, these programs are well-suited for PUI faculty. EPSCoR grants are smaller than federal grants but also less competitive. They are enough to pay summer students, fund materials and travel, and even get some faculty summer pay.

## What Does Success Look Like at a PUI?

Criteria used to evaluate professional development and research achievement at PUIs are similar to Ph.D.-granting institutions: peer-reviewed manuscripts in reputable journals, conference presentations, funded grants, awards. Research output, however, is not similar. It is very difficult to churn out research papers due to teaching and service demands. On average, my group publishes one peer-reviewed manuscript per year. This would be considered unproductive at most Ph.D.-granting institutions, but more than meets expectations at my PUI. Tenure and promotion expectations may state a minimum number of papers; I strived to satisfy this requirement while emphasizing high-quality work and student training. Journal impact factors were requested for my tenure packet but were not important. External letters of evaluation, in contrast, were valued highly.

Additional criteria for success include student awards (e.g., Goldwater Scholarships) and matriculations into graduate programs (Ph.D., M.S., M.D., D.D.S., Pharm.D., etc.), internships, and/or industry positions. Preparing students for successful careers is inherent to the PUI mission. I take great pride in watching my former students progress in their STEM or health careers.

Perhaps most important, there is an intrinsic element to success. After nine years at my institution, I am excited to go into work each day. I still thoroughly enjoy teaching and mentoring undergraduates. Watching them grow as scientists and people is more rewarding to me than any paper or grant. I imagine that many of my PUI colleagues would say the same.

## Conclusion

Tenure-track positions at PUIs are demanding, but in different ways than those at Ph.D.-granting institutions. Faculty spend lots of time teaching, training undergraduates in the lab, and supporting students pursuing careers in STEM and health fields. It is possible to build an impactful research program at a PUI; however, progress can be slow and emphasizes trainee development over output. Working at a PUI can be a highly fulfilling career.

## Notes

Opinions expressed in this manuscript are solely of the author and not of any department, institution, organization, or funding agency.
